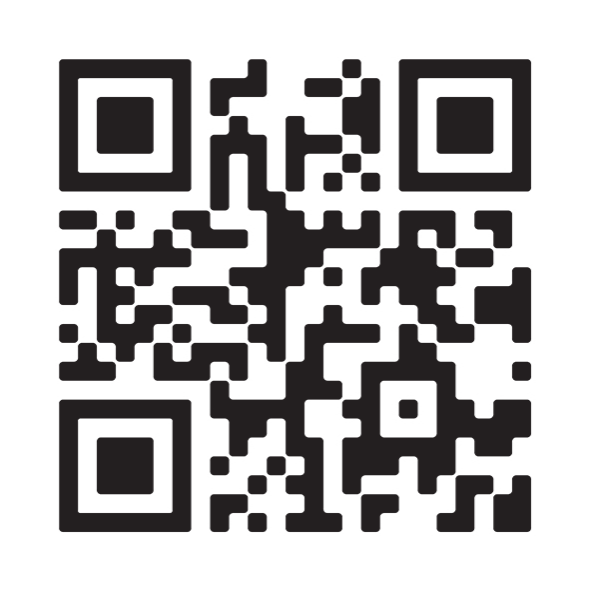# A proof of concept that experience-based management of endometriosis can complement evidence-based guidelines

**DOI:** 10.52054/FVVO.15.3.094

**Published:** 2023-09-24

**Authors:** A Wattiez, L Schindler, A Ussia, R Campo, J Keckstein, G Grimbizis, C Exacoustos, W Kondo, C Nezhat, M Canis, R.L. de Wilde, C Miller, A Fazel, B Rabischong, A Graziottin, P.R. Koninckx

**Affiliations:** Latifa Hospital Dubai, UAE; Prof. Department of Obstetrics and Gynaecology, University of Strasbourg, France; Bourn Hall Fertility Clinic- Mediclinic Dubai; Consultant Università Cattolica, Dpt OBGYN Roma Italy; Life Expertcenter, Leuven, Belgium; Endometriosis Centre, Dres. Keckstein- Villach, Austria and University Ulm, Dpt ObGyn Ulm, Germany; 1st Department of Obstetrics and Gynecology, Aristotle University of Thessaloniki, Greece; Associate Professor of Obstetrics and Gynaecology at the University of Rome Tor Vergata, Italy; Centro Avançado de Cirurgia Ginecológica, Curitiba, Brazil; Nezhat Medical Center, Atlanta GA; Department Obstetrics gynaecology, CHU Estaing, Clermont-Ferrand, France; University Hospital for Gynecology, University Medicine Oldenburg, Carl von Ossietzky University, Germany; Professor, Obstetrics and Gynecology, Department of Clinical Sciences, Rosalind Franklin University of Medicine and Science, North Chicago, IL USA. Director, Minimally Invasive Gynecologic Surgery, Advocate Lutheran General Hospital, Park Ridge, IL USA; Assistant Professor, Obstetrics and Gynecology and Inserm U 1275, CAP Paris-Tech Hôpital Lariboisière, Paris, France; Gynaecological Surgery Department CHU Estaing, Clermont-Ferrand, France; Consultant Professor, University of Verona, Italy, Lecturer, Federico II University, Naples, Italy; Director, Center of Gynecology and Medical Sexology H. San Raffaele Resnati, Milan, Italy; Professor emeritus ObGyn KULeuven Belgium, University of Oxford, and Hon Consultant, UK, University Cattolica, Roma, Italy and Moscow State Univ, Russia

**Keywords:** Experience-based, evidence-based, endometriosis, guidelines, Bayesian, endometriosis management

## Abstract

**Background:**

Management of endometriosis should be based on the best available evidence. The pyramid of evidence reflects unbiased observations analysed with traditional statistics. Evidence-based medicine (EBM) is the clinical interpretation of these data by experts. Unfortunately, traditional statistical inference can refute but cannot confirm a hypothesis and clinical experience is considered a personal opinion.

**Objectives:**

A proof of concept to document clinical experience by considering each diagnosis and treatment as an experiment with an outcome, which is used to update subsequent management.

**Material and Methods:**

Experience and knowledge-based questions were answered on a 0 to 10 visual analogue scale (VAS) by surgery-oriented clinicians with experience of > 50 surgeries for endometriosis.

**Results:**

The answers reflect the collective clinical experience of managing >10.000 women with endometriosis. Experience-based management was overall comparable as approved by >75% of answers rated ≥ 8/10 VAS. Knowledge-based management was more variable, reflecting debated issues and differences between experts and non-experts.

**Conclusions:**

The collective experience-based management of those with endometriosis is similar for surgery-oriented clinicians. Results do not conflict with EBM and are a Bayesian prior, to be confirmed, refuted or updated by further observations.

**What is new?:**

Collective experience-based management can be measured and is more than a personal opinion. This might extend EBM trial results to the entire population and add data difficult to obtain in RCTs, such as many aspects of surgery.

## Introduction

Endometriosis is an enigmatic disease associated with pain and infertility, and pathophysiology, diagnosis and therapy remain debated. Hypotheses on pathophysiology vary from implantation of endometrium after retrograde menstruation (Sampson, 1921; Sampson, 1940) or transport by the bloodstream, to genetic or epigenetic (G-E) ‘incidents’ in endometrial or precursor cells (Koninckx et al., 2019) leading to immunologic changes. The controversy can be summarised as follows: normal (endometrial) cells can develop abnormally because of the environment of the peritoneal cavity and inherited G-E changes in endometrial or immune cells. Alternatively, the endometriosis cell is an abnormal cell that starts developing after new G-E changes are added to the inherited ones, with immunology as a possible favouring factor. Considering the uncertain pathophysiology, the various clinical manifestations of endometriosis and the absence of an adequate animal model and a non-invasive diagnosis, solid evidence is limited, and many management aspects remain debated (Koninckx et al., 2022).

Diagnosis and therapy should be based on the best available evidence. This concept led to the development of evidence-based medicine (EBM) (Barnett and Stagnaro, 1998), resulting in a pyramid of evidence (Djulbegovic and Guyatt, 2017) with randomised controlled trials (RCTs) (Lawrence and Force, 1989) designed to avoid bias, on top of the pyramid. Later, meta-analysis and systematic reviews interpreting the data were added. Despite successful demonstrations of the importance of EBM, clinical implementation has been a challenge (Murad et al., 2016; Farquhar, 2018). Therefore, grades of evidence were added, reflecting the poorly defined interpretation of direct and indirect evidence by a group of experts. However, also the results of an RCT can be problematic if only allocation bias is taken into account by randomisation but not evaluation bias when blinding is not possible and when power is insufficient for rare or multivariate events. As a consequence, RCT data relating to the management of endometriosis remain limited since medical therapy can rarely be blinded, surgery combines low numbers with multi variability, and complications are rare (Koninckx et al., 2022).

Each management of a woman with endometriosis can be considered a unique experiment of diagnosis and treatment with an outcome, which is a complex and multivariate process. However, the clinician will instinctively continue successful management and update or change less successful ones. This progressive update of knowledge by new data to predict the future is fundamental in Bayesian statistics (Lesaffre and Lawson, 2012). Similarly, experience in managing endometriosis is updated by the result of each new management. However, in EBM and the pyramid of evidence, the experience of the individual clinician has been considered a personal opinion of low value (Djulbegovic and Guyatt, 2017) because of the many potential biases. However, a similar collective experience of many clinicians, decreasing personal bias, has more value than a personal opinion.

Over the last decades, traditional statistical analysis with significances and p-values (Fisher 1925; Neyman and Pearson, 1928) has been complemented with Bayesian statistics. The null hypothesis of traditional or frequentist statistics is that there is no difference between groups. The analysis evaluates the probability that an eventual difference can be explained by chance, and a probability of less than 5% is considered significant. Traditional statistical analysis thus can only refute but cannot confirm a hypothesis (Wasserstein and Lazar, 2016). This mistake, frequently made in biomedical research, is called the P-value fallacy (Goodman, 2001). Bayesian statistics, on the contrary, explores the probability that a hypothesis or an observed difference is true, using new data to update all previous data (the prior) (Lesaffre and Lawson, 2012). Bayesian statistics emphasises the uncertainty of whether a hypothesis is correct. Frequentist and Bayesian analyses are related, and a p-value of 0.05 increases the probability of truth from 50% to some 70% (Nuzzo, 2014).

Therefore, to evaluate experience, a group of clinicians with experience in treating endometriosis were asked how they managed some aspects of the disease. Since management is based upon knowledge and a progressive updated experience by learning from the past, we planned to estimate the similarity of experiences in a group of clinicians. Without personal observer bias, this collective experience, based on all previous treatments, literature and discussions, will probably have more value than a personal opinion. The estimation of collective experiences was conceived as a proof of concept to establish a Bayesian prior, permitting subsequent statistical updates and the calculation of the probability that the statements are true.

## Materials and Methods

### The study

The aim of this study was a proof of concept to estimate the collective experience of clinicians. Each diagnosis and treatment can be considered an ‘experiment’ with an outcome, which the clinician uses to update his/her management. This individual experience is considered a personal opinion because of the many potential uncontrolled biases. However, if many clinicians have a similar personal experience, the value of their collective experience is more than a personal opinion. To estimate this collective experience, a group of clinicians were asked to rate their agreement with statements or questions about the clinical management of endometriosis on a 0 to 10 visual analogue scale (VAS). The answers varied between never-always, no-very much, always A-always B (if two alternatives) as explained in the Jotform questionnaires. The collective experience was the sum of the individual experiences, defined by their years of practice and the number of patients treated yearly. The cumulative experiences of those scoring VAS 0 to 10 permitted calculating the frequency distribution of experience for each VAS score.

The questions were organised online using Jotform, an online form builder application. Besides the questions, the respondents were asked about their speciality, gender, age and nationality. Email addresses were used to prevent duplicate answers. After completion of the polls, all data permitting identification were removed to comply with privacy rules.

AW and PK marked questions and conclusions experienced-based if previous experience influenced the management of the surgery- oriented clinicians; they were deemed knowledge- based if management was mainly influenced by information from the literature. Unfortunately, this difference can vary between subspecialists and statements about IVF can be experience-based for infertility specialists and knowledge-based for surgery-oriented clinicians. Experience-based items include issues that are difficult to explore in RCTs (e.g. complications of surgery) and can extrapolate EBM conclusions on a study group to the general population. Knowledge-based items often reflect the knowledge of experts and evaluate the understanding of responders.

### The questionnaires

For a 3-day meeting following an IRCAD training course on endometriosis surgery in Strasbourg in September 2022 (ESGE, 2022), 16 well-known speakers at surgical meetings were asked to organise a workshop within their area of expertise in diagnosing and treating endometriosis. Instead of discussing new and controversial issues, they were suggested to focus on topics clinicians agreed upon and formulate questions for the participants as a preparation for the meeting (to view the questions (MIS Academy, 2023) or conclusions (MIS Academy, 2022)). After several limited tryouts, we (AW, PK) decided to use an online Jotform questionnaire and limit questions to 10 for each workshop in order to keep the total time for answering below 30 min. Subsequently, questions/ statements were polished to be neutral, to avoid complexity, and to fit a visual analogue 0 to 10 scale (VAS). Notwithstanding many emails and internet meetings, this wasn’t easy because of the variable interests of the experts, which explains that the many questions reflect the learning curve while being a compromise between the organisers and the experts.

Unfortunately, we only realised during analysis that this had resulted in experience-based, predominantly knowledge-based, and rather organisational/political-oriented questions and conclusions as judged afterwards by AW and PK. Although the initial aim was to use the answers as a preparation for the workshops and to have the same questions/statements answered after the meeting to judge the impact of the meeting, it was decided to formulate the conclusions of each workshop after discussion with all participants as a second questionnaire.

### The respondents

The first and second questionnaires were answered by those planning to, or attending, the meeting. In addition to the questions or conclusions, responders could identify themselves as gynaecologists with their eventual subspecialty, as surgeons, general practitioners, psychiatrists/psychologists, pain specialists, nutritionists, researchers or persons with lived experience of endometriosis. We registered how they judged their experience in imaging, medical therapy and surgery on a 0 to 10 VAS scale.

### Data analysis

Analysis of the answers was done with SAS (Inc, 2020), and the answers were corrected for the individual experience. The individual experience was the number of women with endometriosis treated, which is the years of practice multiplied by the number of patients treated yearly. The individual experiences permitted calculating the total experience as the sum of all individual experiences. Similarly, the sum of experiences of each VAS scoring permitted the frequency distribution of experience for each VAS score of each statement. These graphs oriented the decision to choose a cut-off as high as ≥ 8/10 VAS for approval and to calculate the sum of the individual experiences approving a statement.

The approval rate of each statement, thus, is the sum of individual experiences of those scoring ≥ 8/10 VAS, divided by the total experience, giving more weight to those with more experience. For each question or conclusion, an eventual effect of gender or personal experience on the VAS answers was evaluated using Spearman correlation or chi-square. Unless indicated otherwise, means and 10th-90th percentile and p-values were calculated using Wilcoxon signed rank test (Koninckx et al., 2023).

## Results

An initial exploratory analysis showed that the large majority of respondents were surgery- oriented and that the number of subspecialists in imaging, urogynecology, fertility or oncology, and the numbers of surgeons, psychiatrists, pain specialists, nutritionists, researchers or those with endometriosis was too low for a significant separate analysis. Therefore, the analysis was limited to surgery-oriented respondents (specialists in minimally invasive surgery ≥ 7/10), with a minimum experience of having managed more than 50 women with endometriosis.

**Figure 1:**
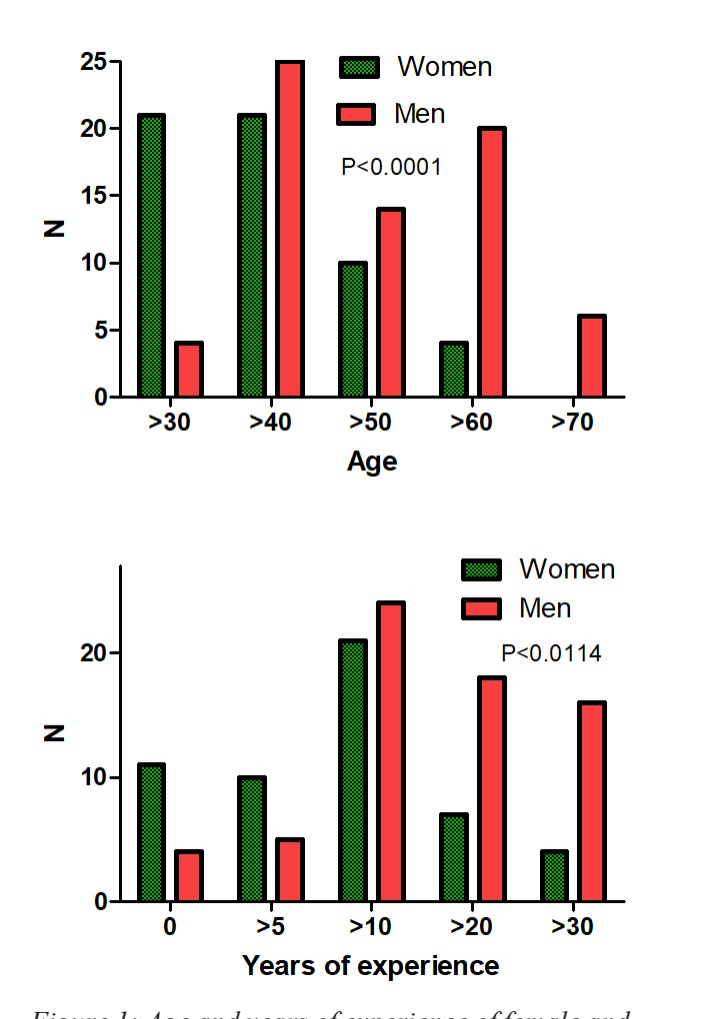
Age and years of experience of female and male surgically oriented responders.

**Figure 2:**
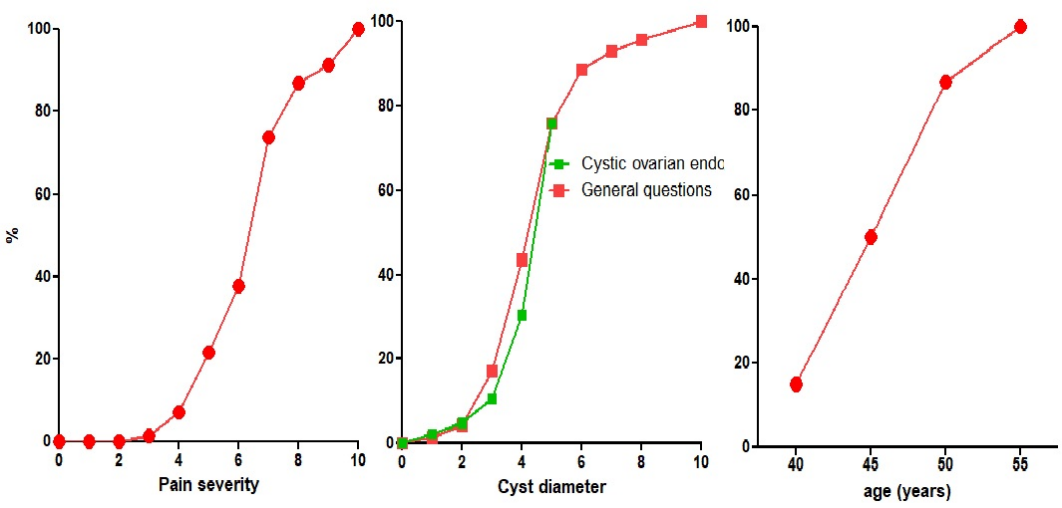
Indications for surgery are multivariate with at least severity of pain, the diameter of the cyst, age of the women, CA125 and fertility. Depicted below are the severity of pain when the only symptom was pain, or the cyst diameter if no pain (red – general question) or little pain (green - question in the workshop ‘cystic ovarian endometriosis’. Since the latter ques- tion had limited the size to 5cm, the responses were normalised to fit a 5cm diameter with a cumulative frequency of 71%. The third graph illustrates the percentage of adnexectomies at different ages for a 4cm endometrioma and a CA125 of 50 IU.

**Figure 3:**
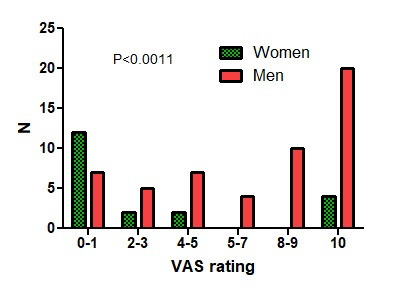
Gender differences (p<0.0011) in answering the question ‘I believe that It is ok when less experienced surgeons do a laparoscopy and refer if too difficult’.

The first questionnaire was answered 134 times by 79 specialists in minimally invasive surgery with a total experience greater than 50, 100, 150 and 200 by 27%, 26%, 10% and 16%, respectively. Of these, 18 had identified themselves as general gynaecologists, five as specialists in imaging, 13 as infertility specialists, seven as oncologists and two as surgeons. The conclusions were answered by 70 with a total experience greater than 50, 100, 150 and 200 by 26%, 22%, 8% and 14%, respectively. Men were older (P<0.0001) than women and thus had more years of experience (P=0.0114) (Figure 1). Age and total experience were too strongly associated to be analysed separately. Ultrasound exams were done by themselves in 62%, but 38% preferred to refer to a specialist. They considered themselves specialists ( ≥ 7/10 VAS) in ultrasonography in 65%, in medical therapy in 92%, in superficial endometriosis or cystic ovarian surgery in 90% and deep endometriosis surgery in 74%. Women were slightly more experienced than men in medical therapy (P=0.0492) but less in superficial (P=0.0253), cystic ovarian (P=0.0173) and deep endometriosis (P=0.0024) surgery. The questionnaire before the meeting (Table I) represented the collective experience of treating 9441 and the conclusions after the meeting (Table II) of treating 11641 women with endometriosis. The indication for surgery of ovarian cysts varied with pain, the diameter of the cyst, age of the woman, CA125, infertility, pain radiation and the suspicion of adhesions by ultrasound. In women complaining of pain only, surgery was rarely performed if pain severity was less than 4/10 and almost always if more than 7/10. For cystic ovarian endometriosis without pain, surgery was seldom performed if the diameter was less than 4 cm and almost always if more than 6 cm (Figure 2). Additional arguments for surgery (≥8/10 VAS) were pain radiation to the anterior side of the upper leg by 42% and to the perineum by 46%, and the suspicion of adhesions to the bowel by 38% and to the ovary by 32%. Excision of cystic ovarian endometriosis was judged difficult surgery, requiring an expert surgeon by 83%. Information on complications between 0.5 and 1% and more than 1% was judged necessary by 65% and 90%, respectively. Results did not vary with the experience or gender of the respondents. Only the statement ‘I believe that It is ok when less experienced surgeons do a laparoscopy and refer if too difficult’ had a biphasic answer, i.e. either approving (≥8/10) or opposing (≤2/10), and surprisingly more women than men were opposing (P<0.0011) (Figure 3).

**Table I:** Questions formulated by the chairpersons of each workshop before the meeting. The individual experience is the number of women with endometriosis treated, or the ‘years of practice’ times ‘the number of endometriosis patients treated/year’. The total experience (TE) is the sum of the individual experience. The approval rate (%) by those with an individual experience of more than 50 women is the sum of their individual experiences approving (defined as ≥ 8/10 VAS) over their total experience. The approval rate (%) thus gives more weight to those with more experience. Questions judged experience-based are indicated in red, knowledge-based in white, and ambiguous or political in blue.

	%	TE
Adolescence		
If after 3 months of medical therapy for pain, pain or dysmenorrhoea is still 3/10 I do a laparoscopy	22	8976
Do you think endometriosis can grow significantly during medical therapy	29	8976
A delay in diagnosis of more than 5 years results in more severe endometriosis lesions	64	8766
Fertility		
I consider and discuss oocyte preservation, before surgery for severe endometriosis in a 30-year-old woman planning a pregnancy.	63	9224
I think the presence of deep endometriosis decreases the success rate of IVF.	60	9336
In young infertile women without pain and no other infertility factors, including the husband, laparoscopy should be done before starting IVF	34	9336
Controlled ovarian stimulation accelerates the growth of endometriosis	28	9336
The presence of a recto-vaginal nodule of 2cm is a contra-indication for IVF	12	9232
Classification		
Which classifications do you use regularly for endometriosis? (tick as many answers as you can)	see text	
The Enzian/#Enzian classification reflect accurately the difficulty of surgery	56	8580
The Enzian/#Enzian classification correlates well with the symptoms of the patient	23	8680
The rASRM classification reflect accurately the difficulty of surgery	5	8524
The rASRM classification correlates well with the symptoms of the patient	4	8524
The AAGL classification correlates well with the symptoms of the patient	11	7118
We need a classification of non-invasive diagnosis	74	8840
An endometriosis classification is useful for IVF treatment	40	8958
Adenomyosis		
In infertile women, a thickened junctional zone needs medical management	31	9011
When adenomyosis is suspected on ultrasound, MRI is needed before surgery	35	9336
If infertility and an adenomyosis nodule of >1cm, I do surgery	89	9252
A classification system for adenomyosis is useful for clinical management	54	8965
Infertility of >5 years and only adenomyosis: I expect the fertility rate after surgery (%)	see text	
For severe diffuse adenomyosis, the complete removal of the longitudinal muscle layer of the myometrium (neometra) is safe if the patient wishes to become pregnant.	4	9252
Sub-endometrial adenomyotic cysts of < 1cm should be treated via hysteroscopy	56	9272
Imaging		
My decision to do a bowel resection is based on ultrasound and or MRI imaging	45	8681
A descriptive imaging report has more value than a classification system	53	9230
Ultrasound imaging accurately predicts ureter involvement even without hydronephrosis	31	9162
In women with cystic ovarian endometriosis larger than 3cm, the ovarian reserve needs to be evaluated e.g. by an antral follicle count (AFC)	70	9129
In adolescents, transabdominal ultrasound can replace transvaginal or transrectal ultrasound	14	9186
Has MRI added value for rectal endometriosis compared with an expert transvaginal ultrasound?	19	9336
An endometrioma of 4cm AND CA125 = 50: from what age do you do an adnexectomy	see text	
How do you describe the size of deep endometriosis?	see text	
Bowel stenosis of the rectum can be diagnosed reliably by ultrasound.	23	9240
Depth of infiltration in the bowel wall can be diagnosed reliably by ultrasound	51	8970
The bowel		
In deep endometriosis, should we excise fibrosis or can we leave it?	54	9177
Is it better to do a double discoid excision (if feasible) over a bowel resection	51	8517
Do you use omental flaps to protect the bowel or ureter after re-anastomosis?	25	8526
Should the type of surgical intervention be decided before surgery (versus during surgery)	47	8967
Do you use daily C reactive protein (CRP) after surgery for deep endometriosis?	52	8429
Should asymptomatic women with a 2*2*2cm bowel nodule be operated on?	7	9113
The ureter		
Ultrasound is an appropriate imaging modality to diagnose hydronephrosis.	90	9240
If hydronephrosis is present, I order a functional assessment of the kidney before surgery.	87	9240
A kidney is considered irrecuperable if renal function is below ..... %	see text	
I am comfortable placing ureteral stents without the assistance of a urologist.	36	8421
Before extensive ureterolysis without hydronephrosis, a ureteral stent is indicated.	32	8652
For how many years do you check for ureter stenosis after reanastomosis	see text	
The right surgeon for each patent		
The future of endometriosis surgery will be a pelvic surgeon (versus a multidisciplinary team )	44	9336
A thin-walled hydrosalpinx should be operated by salpingostomy (versus a salpingectomy).	0	8344
I ask a colorectal surgeon for help to do a bowel resection of the sigmoid.	77	8638
I ask a colorectal surgeon for help to do a bowel resection of the low rectum.	84	8638
My colorectal surgeon agrees that endometriosis requires less extensive surgery than cancer and that a colostomy or ileostomy is not needed	61	8352
I manage my postoperative bowel complication without the assistance of a colorectal surgeon	13	8515
I manage my postoperative ureter complications without the assistance of a urologist	21	8638
I do all ureter surgery without the help of a urologist	34	8638
Sacral root surgery requires the assistance of a neurosurgeon	24	8171
Fertility surgery requires specific training besides severe endometriosis training	62	9049
An endometriosis referral surgeon should operate more than ....... cases a year with bowel, bladder or ureter involvement	see text/fig	
Patient-centered outcomes		
Negative findings during clinical exam and imaging (ultrasound and/or MRI) do not rule out endometriosis	88	9273
Negative findings during laparoscopy do not rule out endometriosis	52	9273
A diagnosis of endometriosis validates symptoms and provides access to relevant care	80	9016
All decisions on endometriosis management should be made together with the patient	93	9273
Educational programs should be added to school curriculums to understand a normal period and menstrual well-being.	91	9273
National healthcare systems should care for and meet the needs of those with endometriosis to receive high-quality and holistic care.	89	9273
Those with endometriosis should be supported to succeed in employment through appropriate adaptions and workplace policies	73	9196
Those living with endometriosis should be involved in setting priorities for endometriosis research and contribute to protocol development	78	9066
Adhesion prevention		
Incomplete endometriosis excision causes more adhesions	53	9336
Barriers should be used systematically after endometriosis surgery	40	8916
I estimate that barriers decrease postoperative adhesions by .....(%)	see text	
After excision of cystic ovarian endometriosis, adhesions occur in (%)	see text	
Cystic ovarian endometriosis		
What size endometrioma in a 25-year-old patient with little pain requires surgery	Fig 1	
The capsula of an endometrioma is fibrosis and not endometriosis	40	8916
Tick sequentially your first and second choice to treat endometriomas of 7-8 cm in a 25-nulliparous patient	see text	
Tick sequentially your first and second choice to treat endometriomas of 3-4 cm in a 25-nulliparous patient	see text	
What is your preferred method to achieve hemostasis after excision?	see text	
Femtech AI		
All laparoscopic diagnoses of endometriosis must be confirmed by pathology	40	8916
An app would improve the follow-up after medical or surgical treatment of endometriosis	60	9124
An app would improve the diagnosis of endometriosis	49	8629
Indocyanine green should be used to check vascularisation after bowel resection-anastomosis	33	8251
Indocyanine green should be used to check vascularisation after ureter resection-anastomosis	39	8121
For the excision of deep endometriosis with hydronephrosis, robotic surgery is superior	15	8505
Nerves		
Severe menstrual sciatalgia with a normal MRI needs a surgical exploration of the sciatic nerve	24	8103
An image of the dermatomes should be used in the medical records	56	8210
The surgeon needs to know the dermatomes of The genito-femoral nerve	77	7998
The ilio inguinal nerve	80	7536
The ilio hypogastric nerve	82	7788
The sciatic nerve	85	7998
Pain		
Deep endometriosis without pain or dyspareunia decreases libido	14	9084
Chronic pelvic pain without dyspareunia decreases libido	72	9084
Centralization of chronic pain occurs after how many months?	see text	
Pain reduction after surgery should be judged after how many months?	see text	
The memory of pain makes the pain worse when there is a recurrence of endometriosis.	68	9277
Sexuality	####	0
When suspected or confirmed endometriosis, should we ask about deep dyspareunia	96	9093
When suspected or confirmed endometriosis, should we ask about comorbid bladder symptoms, “the evil twins” (post-coital/recurrent cystitis, urinary urgency and/or frequency?)	90	9093
When suspected or confirmed endometriosis, should we ask about vulvar pain and/or introital dyspareunia?	77	8937
Do you use a validated questionnaire (e.g. FSFI -Female Sexual Functioning Index) to judge the impact of endometriosis on sexual function	15	8799
When suspected or confirmed endometriosis, do you ask about the sexuality of the couple including the partner	58	9093
Medical therapy		
During medical treatment, I do a 6 or 12-monthly ultrasonographic follow-up	72	9336
In adolescents with dysmenorrhoea >7/10 and negative exams, I start medical treatment	86	9336
I use medical treatment before surgery for deep endometriosis	38	9336
I use medical treatment before surgery for cystic ovarian endometriosis	30	9240
After endometriosis surgery, I give medical therapy without menstruation until pregnancy wish	12	8849
I estimate the placebo effect of medical treatment for pelvic pain at	see text	

**Table II:** Conclusions formulated by the chairpersons of each workshop after the meeting and discussion with the participants. The individual experience is the number of women with endometriosis treated, or the ‘years of practice’ times ‘the number of endometriosis patients treated/year’. The total experience (TE) is the sum of the individual experience. The approval rate (%) by those with an individual experience of more than 50 women is the sum of their individual experiences approving (defined as ≥ 8/10 VAS) over their total experience. The approval rate (%) thus gives more weight to those with more experience. Questions judged experience-based are indicated in red, knowledge-based in white, and ambiguous or political in blue.

Conclusions of the consensus workshop in Strasbourg	%	TE
Adolescence	approval	
Adolescent girls with an enhanced hereditary risk of developing endometriosis should be informed about potential prevention by antioxidants such as fruit and vegetables and a healthy lifestyle from menarche onwards.	51.4	9251
All vaginitis should be treated adequately.	79.1	9251
Adolescents with severe dysmenorrhoea (>7/10, eventually >5/10) deserve an ultrasound or MRI investigation, and if negative, a trial therapy with continuous estro-progestagens.	85.7	9251
Since, during medical treatment, some (10-30%) endometriosis lesions can progress, a yearly follow-up by imaging is recommended.	79.6	8851
Fertility considerations		
Cryopreservation of oocytes should be discussed with all endometriosis patients.	46.6	9251
Cryopreservation of oocytes should be proposed for endometriosis patients 30-35 years old.	61.0	9376
Endometriosis does not change the outcome of IVF, the impact being quantitative, not qualitative.	57.4	8614
Endometrioma with associated symptoms ( Pain >7/10, hydrosalpinx etc.) is an indication for surgery.	93.2	8704
IVF can be offered as a first-line treatment in all endometriomas without associated symptoms	42.4	8851
IVF outcomes are similar in endometriosis patients with agonist or antagonist protocol	63.4	8658
The recurrence rates of endometriosis are not increased by ART stimulation.	54.6	8557
Women with major pain or infertility and deep endometriosis require competent surgery.	89.3	8957
Endometriosis classifications		
A classification for endometriosis should comprise deep endometriosis and adenomyosis as separate entities.	82.9	8557
Information on the localisation and extension of DE is important for planning surgery.	91.6	8957
A classification needs to be validated by predicting symptoms and/or the outcome of treatment.	73.0	8957
#Enzian describes the location and extension of deep endometriosis, whether evaluated during surgery or by imaging	95.0	8959
The description of superficial and cystic ovarian endometriosis is consistent in all classifications, including #Enzian.	60.8	8480
Adenomyosis		
Adenomyosis is defined as the presence of endometrial glands in the myometrium with or without surrounding smooth muscle hyperplasia.	82.5	8557
Thickening of the inner myometrium (junctional zone) alone should not be considered adenomyosis.	82.5	8557
Thickening of the inner myometrium (junctional zone) alone might be a different entity of adenomyosis, only important for reproduction.	57.9	8657
Strict ultrasound criteria as defined by the MUSA group (hyperechoic areas, cystic anechoic spots and linear striations) are necessary for the diagnosis of adenomyosis.	81.4	8557
Imaging reports of adenomyosis should describe whether local or diffuse, cystic or solid and the size, extent and location.	97.3	8557
IVF and adenomyosis: cryopreservation and embryo transfer after down-regulation is recommended	70.5	8557
Surgery is indicated only for focal or cystic adenomyosis.	76.1	11608
Imaging		
Symptoms, clinical exam and TV-US, are the first line of investigation of pelvic endometriosis.	98.0	8957
Imaging is important for the management of pelvic endometriosis and for counselling the patient.	87.9	8957
A description is more complete than a classification, and a concise standard template would be useful.	92.9	8207
The value of imaging is limited to explore large somatic nerve involvement.	79.1	8557
Diagnostic accuracy should consider prevalence, being higher in dedicated centres.	88.7	8461
TVU and MRI require knowledge of endometriosis	97.1	4208
The bowel		
Surgery is not indicated for asymptomatic endometriosis, i.e.no, pain, no infertility, and no bowel or ureter stenosis.	85.3	8557
Deep endometriosis with pain >4/10, not responding to medical treatment, requires surgery.	85.9	8557
The type of intervention that needs to be performed for deep endometriosis is decided during surgery.	54.4	8557
“Visual” disease-free margins are sufficient for deep endometriosis surgery.	81.5	8557
Segmental bowel resection for deep endometriosis requires a transmesorectal approach.	84.4	8480
The preferred surgery for deep endometriosis of the rectum is excision, eventually completed with segmental resection and a short bowel resection if needed.	66.1	8480
NOSE is useful	47.3	8251
When linear and circular staple lines cross after bowel resection, a suture to prevent leakage is recommended.	79.8	8384
Early oral feeding (ERAS protocol) is recommended.	91.6	8461
Early discharge and CRP follow-up need to be balanced individually.	99.3	8461
The ureter		
US is an appropriate imaging modality to evaluate hydronephrosis.	95.2	8480
In the case of hydronephrosis, functional renal imaging is indicated to assess the percentage of functionality.	97.2	8480
Imaging should be performed after surgical management of endometriotic hydronephrosis (shaving, stitching, resection and anastomosis, or ureteral reimplantation) to rule out stenosis/recurrence at 3 months, then annually for 4 years.	94.0	8480
In patients with loss of renal function without symptoms, nephrectomy is not indicated.	80.1	8384
Laparoscopic management, with or without robotic assistance, of urological endometriosis is feasible and preferable.	96.2	8461
Intrinsic ureteral endometriosis requires the excision of the diseased portion of the ureter.	98.0	8557
The distance of the ureter resection from the bladder does not affect the indication for reimplantation or reanastomosis	44.0	8567
The right surgeon for each patent		
During advanced endometriosis surgery, all skills needed must be present, whether represented by one or more surgeons.	87.9	8957
The endometriosis patient deserves a lead gynaecologist during and after surgery.	87.2	8957
Early identification of those who have the talents to become expert endometriosis surgeons is recommended.	87.5	8880
Home-based surgical training should be evaluated to improve work-life balance.	77.2	8880
In the absence of proven advantages in the outcome, it is the choice of the surgeon to perform classic or robot-assisted laparoscopic surgery	88.7	8957
Patient-centered outcomes		
A diagnosis of endometriosis is important because putting a name to the symptoms gives it validity to the individual, as well as with family and friends, at school and work, and within the healthcare system to gain access to care.	88.9	8957
It is important that a prompt diagnosis is received, using the most appropriate method of diagnosis so that prompt management can commence to reduce the risk of progression, improve health-related quality of life, and preserve fertility. This includes an early working diagnosis.	88.9	8957
Recognising endometriosis requires experience. Without specialist training, endometriosis may not always be recognised; not finding endometriosis during imaging or laparoscopy, therefore, does not always rule out endometriosis as the cause of symptoms.	88.2	8957
The ultimate goal for a patient may not always be pain relief but could be different aspects related to the improvement of quality of life. Therefore, to provide effective treatment, it is important to understand why the person is seeking medical assistance, including for recurrent symptoms.	90.6	8957
Decisions should be made together with the patient. Subsequent treatment and management should be in-terdisciplinary, holistic, and long-term, centred around both symptoms and any subsequent consequences of endometriosis, whether physiological, psychological, or social.	88.1	8966
No one should start a period without being taught about it. Age-appropriate education about what is a normal period and overall menstrual well-being should be part of the school curriculum.	86.9	8957
Governments should ensure the healthcare systems in their country cater for and meet the needs of those with endometriosis to receive high-quality and holistic care.	98.8	8207
Those living with endometriosis should be involved in setting priorities for endometriosis research.	62.2	8880
Investigators should seek the feedback and input of those living with endometriosis at all stages of the research, from the development of the protocol to the delivery of the results.	83.3	8207
To ensure research results are comparable across centres around the world, uniform standards for the collection of clinical data and bio-specimens are crucial.	90.6	8957
Adhesion prevention		
Postoperative adhesions after endometriosis surgery can be reduced by laparoscopic surgery, respecting microsurgical principles.	84.1	9251
The prediction of the risk of adhesions in the individual woman is limited.	79.8	9251
Surgery for severe endometriosis requires adhesion prevention awareness and protocols.	82.5	9251
Cystic ovarian endometriosis		
Although the likelihood of ovarian cancer in a reproductive-age woman is small, the first step in the evaluation of a potential endometrioma is to perform transvaginal ultrasound imaging.	91.6	8957
The use of CA-125 does not distinguish between an endometrioma and ovarian cancer in a reproductive-age woman and, therefore, need not routinely be performed.	74.2	8957
In women complaining of pain at the side of the endometrioma, the pain is more likely secondary to pelvic sidewall endometriosis and adhesive disease. For pain control, the sidewall disease must be removed.	71.3	8957
In a geographic area where ART is routinely performed, asymptomatic women attempting pregnancy with timed intercourse or inseminations should not remove endometrioma (so as to not negatively impact ovarian reserve) even though it may increase the ability of non-ART pregnancy.	56.5	8861
When there is no access to ART or access is limited, consider laparoscopy and ovarian cystectomy to enhance the opportunity for pregnancy.	81.2	8957
The location of the endometrioma is at risk of rupture during egg retrieval, placing the patient at risk of embryo contamination or ovarian abscess.	74.1	8480
The number of antral follicles on the affected side is markedly reduced.	77.3	8480
Despite normalising other factors, there is continued implantation failure.	64.0	8480
Based on location and antral follicle count, endometrioma greater than 5 cm should be removed before or after retrieval.	80.3	7730
Suture repair and the use of bio-coagulants to gain hemostasis may offer less impact on ovarian health.	82.0	8557
Newer treatment techniques, such as PlasmaJet desiccation of the cyst bed, combined CO2laser excision and desiccation at the hilum, and sclerotherapy with EtOH, appear to cause a less negative impact on ovarian health, although additional studies are required.	55.0	8557
In reproductive-age women, part of the discussion regarding ovarian cystectomy for ovarian endometrioma must include egg freezing, ideally prior to surgery, unless pain is acute, the endometrioma position would compromise egg retrieval and/or antral follicle count is markedly reduced.	78.4	8431
Femtech AI		
Clinical trials to test specific biomarkers of endometriosis should be VALUABLE, excluding confounding uterine conditions and inflammatory diseases	83.3	8057
Clinical trials to test sensitive biomarkers of endometriosis should gather a significative number of patients to be used as a SCREENING TEST with a sensibility of 85%.	79.7	8957
Clinical trials to test specific biomarkers of endometriosis should gather a significative number of patients to be used as a DIAGNOSTIC TEST with a specificity of 85%.	75.7	8957
Clinical trials to test specific biomarkers of endometriosis should have VERACITY, being based on proof of endometriosis by pathology	84.0	8747
Clinical trials to test specific biomarkers of endometriosis should carry VARIABLE populations, being multicentric, multiethnic, multi-environmental, multistage and documented studies.	83.3	8747
Robotic surgery shortens the learning curve to treat complex cases of endometriosis	37.2	8957
Robotic surgery makes it easier to perform complex tasks like DIE and Nerve sparing	41.1	8957
Robotic surgery provides better ergonomics for complex surgeries with less fatigue for the surgeon	77.7	8897
Nerves		
The pelvic surgeon needs to have perfect knowledge of descriptive and functional Pelvic Neuroanatomy	85.6	9257
In women with pain, the clinician should realise eventual nerve involvement before surgery	82.1	8957
If symptoms and clinical exam make a nerve involvement likely, a precise diagnosis is needed eventually with the help of a neurologist and before any surgery	73.7	8957
Nerve-sparing is to preserve mostly autonomic nerves with the first factor of success being the surgeon’s awareness of their location	88.2	8957
Systematic full surgical dissection of the nerves is not mandatory without entrapment of nerves by endometriosis.	87.3	8957
Surgery of nerves requires knowledge of rules in nerve dissection, i.e. atraumatic avoiding grasping and traction, and using the right instruments	89.5	8957
Pain		
Early diagnosis and treatment of endometriosis-associated pain is important and may prevent nociplastic pain (central sensitisation).	90.6	8957
Modern pain science should be a core competency for those providing care for women with endometriosis	85.2	8957
Adjunct therapy, such as physiotherapy, yoga and cognitive therapy, can be useful for patients with endometriosis-associated pain	88.2	8957
Sexuality		
Endometriosis-associated pain impairs all aspects of female sexual function through sexual pain.	89.9	8957
Severe dysmenorrhea, heavy menstrual bleeding and deep dyspareunia could be early predictors of endometriosis. For this reason, these symptoms need timely clinical attention.	91.5	8837
Over time cyclic pelvic pain and chronic pelvic pain further complicate endometriosis-associated pain. Dyschezia and dysuria deserve specific attention, although less frequently complained of	91.4	8760
Pelvic floor muscles tonus and tension should be carefully clinically evaluated in every woman complaining of endometriosis. Evidence shows that hyperactive pelvic floor is frequently associated with vestibulodynia, superficial dyspareunia/sexual pain and recurrent cystitis comorbid with endometriosis.	82.0	8760
When symptoms and/or imaging suggest endometriosis, hormonal medical treatment should be considered, in absence of lesions that indicate a surgical treatment. Continuous progestogen, or continuous hormonal contraceptives, or regimens with a reduced number on hormone free intervals (HFI) and periods should be considered as first line.	87.4	8760
After gynecological surgery for endometriosis, hormonal medical treatment with either continuous progestogen or continuous hormonal contraceptives should be considered when pregnancy is not desired.	91.0	8760
Endometriosis is characterised by a progressive pelvic and systemic inflammation. Androgen receptors are present in endometriotic lesions. In vitro, testosterone has shown to reduce tissue inflammation through the significant reduction of pro-inflammatory cytokines and significant increase of anti-inflammatory cytokines. Ongoing studies are aimed at evaluating its anti-inflammatory and pro-sexual potential in vivo, in women with endometriosis and sexual pain disorders and associated FSD.	78.6	8150
Medical therapy		0
Hormonal medical therapy or pregnancy or menopause only inactivate endometriosis.	71.6	8945
During medical treatment some (10-30%) lesions progress. Therefore during treatment, at least a yearly follow-up by ultrasound should be done.	70.6	8725
Except as preparation for IVF, medical treatment does not enhance fertility.	69.3	4712
20-30% of women with endometriosis and pain do not respond to medical therapy	75.4	8900
Placebo therapy of hormonal medical therapy is estimated at 20-30%	81.8	8945
Hormonal medical therapy cannot be blinded in comparison to a placebo’	70.2	8552
Traditional statistics are inappropriate to evaluate hormonal medical therapy because of the variability of endometriosis’	74.5	8855
The maximal efficacy of GnRH agonists and antagonists is similar	72.5	7879
The efficacy of all oestro-progestins is similar	57.1	8725
Hormonal medical treatment to abolish menstruation should be considered/investigated as prevention.	88.2	7975
It is unclear how to define ‘sufficient’ pain relief during medical treatment and whether medical therapy is less effective for older more fibrotic lesions.	64.1	4730

The indication for surgery of ovarian cysts varied with pain, the diameter of the cyst, age of the woman, CA125, infertility, pain radiation and the suspicion of adhesions by ultrasound. In women complaining of pain only, surgery was rarely performed if pain severity was less than 4/10 and almost always if more than 7/10. For cystic ovarian endometriosis without pain, surgery was seldom performed if the diameter was less than 4 cm and almost always if more than 6 cm (Figure 2). Additional arguments for surgery (≥8/10 VAS) were pain radiation to the anterior side of the upper leg by 42% and to the perineum by 46%, and the suspicion of adhesions to the bowel by 38% and to the ovary by 32%. Excision of cystic ovarian endometriosis was judged difficult surgery, requiring an expert surgeon by 83%. Information on complications between 0.5 and 1% and more than 1% was judged necessary by 65% and 90%, respectively. Results did not vary with the experience or gender of the respondents. Only the statement ‘I believe that It is ok when less experienced surgeons do a laparoscopy and refer if too difficult’ had a biphasic answer, i.e. either approving (≥8/10) or opposing (≤2/10), and surprisingly more women than men were opposing (P<0.0011) (Figure 3).

The questions formulated as a preparation for the meeting and their approval rate are listed in Table I, and the conclusions after the meeting in Table II. The tables indicate in red what we judge today as experience-based statements, which, in retrospect, are relevant for this proof of concept. Knowledge-based statements are indicated in white, and ambiguous or statements about the organisation of medical care in blue. The responses will be briefly summarised, considering approved if over 75% of the total experience had scored the VAS answer with 8/10 or more. Statements/questions estimated to be predominantly knowledge-based are indicated as such since, in retrospect, they are not relevant for this proof of concept since they do not reflect experience.

### Adolescence (UA)

#### Conclusions

It was approved that adolescents with severe dysmenorrhoea deserve an ultrasound or MRI investigation and, if negative, a trial therapy with continuous estro-progestagens, that a yearly follow-up by imaging is needed during medical treatment since some endometriosis lesions can progress and that vaginitis needs adequate treatment. However, in retrospect, the question did not permit to conclude that this was based on the microbiome and the G-E pathophysiology of endometriosis. Not approved was that adolescents with a severe hereditary risk could bennefit from preventing endometriosis (Knowledge Based KB).

#### Questionnaire

Before the meeting, only 29% considered that endometriosis could grow during medical therapy (KB), reflecting the effect of the meeting. It was not approved that a delay in diagnosis of more than five years results in more severe endometriosis and that laparoscopy is indicated if, after three months of medical therapy, pain or dysmenorrhoea is still 3/10.

### Fertility considerations (CR)

#### Conclusions

It was approved that a cystic ovarian or deep endometriosis with severe pain or an associated hydrosalpinx is an indication for surgery. The low approval rates of IVF-related statements (KB) illustrate the knowledge gap between fertility experts and surgery-oriented gynaecologists.

#### Questionnaire

This knowledge gap also explains that a rectovaginal nodule of 2*2*2 cm was not approved as a contra-indication for IVF (KB).

### Endometriosis classifications (KJ)

#### Conclusions

It was approved that the #Enzian classification describes all aspects of endometriosis, that Information on the localisation and extension of deep endometriosis is important for planning surgery, and that deep endometriosis and adenomyosis are different entities. Only 73% approved that classifications need to be validated (KB).

#### Questionnaire

The KB questions were not approved, including that a classification needed to be validated by predicting the difficulty of surgery or symptoms. The use of classifications varied widely. If one classification was used, it was the ASRM, Enzian, #Enzian, AAGL and EFI by 12%, 3%, 20%, 5% and 3%, respectively. If two classifications were used, it was the ASRM and Enzian, #Enzian, AAGL or EFI by 9%, 14%, 3% and 2%, respectively. Enzian and AAGL or EFI were used by 3% and 5%, #Enzian and AAGL or EFI by 2% and 3% and AAGL and EFI by 6%, respectively.

### Adenomyosis (GG)

#### Conclusions

It was approved that adenomyosis is defined by pathology, that Morphological Uterus Sonographic Assessment (MUSA) (Harmsen et al., 2019) criteria should be used for ultrasound diagnosis, and that adenomyosis needs to be described as focal or diffuse, cystic or solid, together with the size and location. Also approved was that thickening of the JZ alone should not be considered adenomyosis and that it might represent a different entity important for reproduction (KB), but not that adenomyosis decreases IVF results (KB).

#### Questionnaire

It was approved that infertility with focal or cystic adenomyosis is an indication for surgery. The KB questions were not approved: that a thickened junctional zone needs medical treatment, that MRI before surgery has an added value, and that sub-endometrial adenomyotic cysts of less than 1cm should be treated by hysteroscopy.

### Imaging (EC))

#### Conclusions

It was approved that ultrasound is the first line imaging and that ultrasound, clinical exam, and symptoms are important for diagnosing and managing endometriosis. Also approved was that accuracy in dedicated centres is higher, that knowledge of endometriosis is required for ultrasonography, that the value is limited for exploring large somatic nerves despite expertise, that classification systems are important as a common language and to share results between disciplines, and that a presurgical evaluation needs an accurate anatomical description.

#### Questionnaire

Deep endometriosis nodules were measured using 1, 2 or 3 dimensions by 10%, 23% and 67% of ultrasonographers, respectively. That in women with cystic ovarian endometriosis larger than 3cm, the ovarian reserve needs to be evaluated (KB) was approved by 70% only. It was not approved that ultrasound imaging can predict bowel stenosis, the depth of infiltration in the bowel wall, the need for a bowel resection, ureter involvement in the absence of hydronephrosis, that transabdominal ultrasound can replace transvaginal ultrasound in adolescents and the added value of MRI.

### The bowel (KW)

#### Conclusions

It was approved that asymptomatic endometriosis is not an indication for surgery, that deep endometriosis with pain involving the bowel requires surgery, that segmental bowel resection for deep endometriosis requires a transmesorectal approach, that the preferred surgery is excision, eventually completed by a discoid or bowel resection, that a suture to prevent leakage is recommended when linear and circular staple lines cross, that early oral feeding is the way forward and that the use of C-reactive protein (CRP) and discharge needs individualisation. It was not approved that the type of surgery should be decided during surgery and that transanal natural orifice specimen extraction (NOSE) (Malzoni et al., 2022) is an improvement.

#### Questionnaire

It was approved that an asymptomatic nodule is not an indication for surgery, but not that fibrosis should be excised, that a bowel resection should be decided during surgery, or that double discoid resection or omental flaps or CRP to monitor the postoperative period, are useful.

### The ureter (NC)

#### Conclusions

It was approved that ultrasound evaluation is appropriate to diagnose ureter stenosis, that the renal function needs to be assessed in women with hydronephrosis, that the asymptomatic loss of renal function is not an indication for nephrectomy, that ureter endometriosis can be treated laparoscopically and that intrinsic endometriosis requires excision of the diseased part of the ureter. It was not approved that the distance from the bladder affects the indication for reimplantation or reanastomosis, reflecting the discrepancy with the expert opinion for rare pathologies.

#### Questionnaire

It was approved that ultrasound is appropriate to diagnose hydronephrosis and that renal function needs to be assessed in women with hydronephrosis. It was not approved that a ureter stent should be placed without hydronephrosis and that the gynaecologist should do it. A kidney is considered irrecuperable if the remaining function is less than 5%, 10%, 15%, 20%, 25%, 30% or 35% by 20%, 32%, 26%, 8%,3%, 6%, and 4%, respectively. After a reanastomosis, the ureter is checked for 1, 2, 3, 4, 5, and 10 years by 9%, 28%, 15%, 4%, 27%, and 17%, respectively.

### The right surgeon for each patient (CM)

#### Conclusions

It was approved that the gynaecologist should coordinate endometriosis surgery, whether done by a pelvic surgeon or a multidisciplinary team, that surgical training is important , and that robotic surgery is a choice (KB).

#### Questionnaire

**Figure 4:**
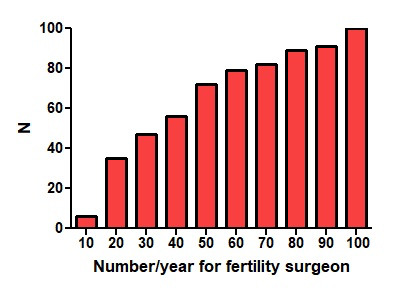
Age and years of experience of female and male surgically oriented responders.

The questions were either informative or knowledge-based. They illustrated that most gynaecologists ask a colorectal surgeon for bowel resections or bowel complications after surgery, that many ask the help of a urologist, and that 62% think that fertility surgery requires specific training. Also, the yearly number of surgeries with bowel, bladder and ureter involvement, which are needed to be an endometriosis referral surgeon, was not clear, ranging between 20 and more than 100 (Figure 4).

### Patient-centred outcomes (HL)

#### Conclusions

That the only goal may not be pain relief, thus requiring understanding the patient for effective treatment was approved. All other knowledge-based statements were approved, such as the importance of an early diagnosis for the patient, the input of the patient and that many other aspects need to be considered besides pain, that governments should organise centres with adequate experience, that education on menstrual well-being should be part of school programs, that uniform standards are needed. Although it was approved that researchers should get feedback from those living with endometriosis, It was not approved that they should set research priorities.

#### Questionnaire

It was approved that negative findings during clinical exam and imaging do not rule out endometriosis and that decisions on endometriosis management should be made together with the patient. Also approved was that educational programs should be added to school curriculums (KB), that appropriate healthcare systems for endometriosis are needed (KB), and that those living with endometriosis should be involved in setting priorities for endometriosis research.

### Adhesion prevention (DR)

#### Conclusions

It was approved that microsurgical principles should be used, that the surgeon should be aware of adhesions, that adhesion prevention protocols are needed, and that the prediction of the adhesion risks in the individual woman is poor (KB).

#### Questionnaire

It was not approved that incomplete endometriosis surgery causes more adhesions or that barriers should be used in endometriosis surgery. The knowledge of the efficacy of barriers was limited and estimated to be less than 20%, 21% to 30%, 31% to 40%, 41% to 50% and over 50% by 31%, 34%, 12%, 17% and 6%, respectively. The risk of adhesions after excision of cystic ovarian endometriosis was estimated at 20%, 40%, 60%, 80% and 100% by 27%, 30%, 21%, 14% and 5%, respectively.

### Cystic ovarian endometriosis (MC)

#### Conclusions

It was approved that diagnosis should start with transvaginal ultrasound, that CA-125 has little added value, that surgery is indicated when the location and size of the endometrioma increase the risk of rupture, and that sutures are preferred for hemostasis. Only 71% considered that sidewall disease under the endometrioma should be removed. It was not approved that newer techniques such as PlasmaJet, combined CO2 laser or excision of the hilus and sclerotherapy were superior to prevent oocyte damage, and questions about the indication of egg freezing or of IVF in asymptomatic women with cystic ovarian endometriosis (KB).

#### Questionnaire

The question about cyst diameters being an indication for surgery was strikingly similar to the answers to the general questions (Figure 1). To treat endometriomas of 7-8 cm in a 25 nulliparous patient, the first choice was excision. The second choice was divided between ethanol sclerosis, plasma jet vaporisation, excision and vaporisation of the hilus or a 2-stage technique. To treat 3-4 cm endometriomas, the first choice was excision or superficial treatment, and the second choice was superficial treatment. The preferred method for hemostasis was bipolar coagulation by 50%, plasma jet by 35%, procoagulants by 10% and suturing by 9%.

### Femtech and artificial intelligence (FA)

#### Conclusions

All statements, being knowledge- based, were endorsed. Trials to test biomarkers deal with big data, requiring the “Five V rule”. Veracity indicates the need to have proof of endometriosis by pathology, especially for small lesions. A Valuable trial has to exclude confounding factors such as inflammatory or immune diseases. The Volume of the tested groups needs to permit over 85% sensitivity and specificity for a screening or diagnostic test, including variable populations, ethnicities, and environments. The Velocity or the translation from biomarker discovery to a feasible, reproducible, cost-effective test was not evaluated.

#### Questionnaire

The superiority of an app for the diagnosis or follow-up of endometriosis of endometriosis, of robotic excision of severe deep endometriosis or of indocyanine green (KB) were not approved.

### Nerves (RB)

#### Conclusions

Results confirm a recent review (Aleksandrov et al., 2022). It was approved that nerve involvement should be evaluated before surgery, that knowledge of descriptive and functional Pelvic Neuroanatomy is needed, that nerve sparing is needed by avoiding autonomic nerves, and that the rules to prevent nerve damage should be known. Not approved is the systematic, complete dissection of nerves, and 72% approved that a precise diagnosis of nerve involvement, eventually with the help of a neurologist, was required before surgery.

Any endometriosis surgeon must know that deep infiltrating endometriosis can affect pelvic somatic nerves, the sacral plexus, the obturator nerve and, more rarely, the femoral nerve. There is also an isolated form of endometriosis of the sciatic nerve, which may be detected preoperatively and can only be confirmed by a specific exploration of the sciatic nerve itself. Therefore, any preoperative history should include questioning of neuropathic somatic pain and neurological disorders with gait disturbances, foot drop and Trendelenburg. In such suspicions, radiologists should specifically pay attention to the eventual involvement of the somatic pelvic nerves.

#### Questionnaire

It was approved that an endometriosis surgeon should know the dermatomes of the genito- femoral, the ilioinguinal, the iliohypogastric and the sciatic nerve (KB). It was not approved that images of dermatomes should be used in the medical record and that the sciatic nerve should be explored in women with severe menstrual sciatalgia and a normal MRI.

### Pain (AS)

#### Conclusions

It was approved that adjunct therapy, such as physiotherapy, yoga and cognitive therapy, can be beneficial for patients with endometriosis- associated pain, that early diagnosis and treatment are essential to prevent nociplastic pain (KB) (centralisation of pain) and that modern pain science should be a core competency for those providing care for women with endometriosis (KB).

#### Questionnaire

It was not approved that memory of pain worsens the pain in women with a recurrence of endometriosis (KB) and that chronic pelvic pain without dyspareunia decreases libido (KB). Centralisation of pain was estimated to occur after 6 and 12 months by 64% and 100%, respectively, which is in line with 50% and 84% estimating that the effect of surgery on pain can be judged after 3 or 6 months, respectively.

### Sexuality (GA)

#### Conclusions

It was approved that endometriosis- associated pain impairs all aspects of female sexual function, that deep dyspareunia is the most potent blocker of genital arousal and vaginal lubrication, that, therefore, early diagnosis of endometriosis is important, and that pelvic floor muscle tonus should be clinically evaluated because of the association of pelvic floor hyperactivity with sexual pain, vestibulodynia, and recurrent post-coital cystitis.

#### Questionnaire

Only 77% considered that women suspected of having deep endometriosis should be asked about comorbid bladder symptoms, vulvar pain and introital dyspareunia. Not approved was the systematic use of a validated score for sexual functioning, such as the female sexuality functioning index (FSFI), or of the exploration of the sexuality of the partner.

### Medical treatment (PK)

#### Conclusions

It was approved that medical therapy has a placebo effect of 20% to 30%, and 20% to 30% of women with endometriosis do not respond. Not approved were knowledge-based statements that endometriosis lesions may progress during medical treatment, that medical therapy does not enhance fertility, that medical therapy cannot be blinded, that the efficacy of all oestro-progestins is similar, that hormonal medical therapy or pregnancy or menopause only inactivate endometriosis and that traditional statistics are inappropriate to evaluate hormonal medical therapy because of the variability of endometriosis.

#### Questionnaire

It was approved that endometriosis needs ultrasonographic follow-up during medical treatment, that adolescents with severe pain but a negative exam deserve medical treatment and that medical treatment before or after surgery is not useful. The placebo effect of medical therapy was estimated at 10%, 20% or 30% by 25%, 29% and 40%, respectively.

## Discussion

These data are a proof of concept to document collective experience in managing endometriosis. The results are a first estimation, or a Bayesian prior, in a limited group of surgery-oriented clinicians. The results, as listed in Table I (before the meeting) and Table II (after the meeting), will need to be updated, refined and completed with new data permitting to judge the value of the conclusions. Results also need to be completed with data from other sub-specialists, and any analysis will need to be detailed, taking into account the type and quantity of the experience and the exact value of the answers, which we limited to ≥75% of the total experience giving a VAS score of 8/10 or more. Therefore, only summaries of the collective experience after diagnosing and treating nearly 10.000 women with endometriosis by surgery-oriented gynaecologists are given without discussing the results. The topics covered are limited to the ten conclusions or questions for each of the 16 workshops, and conclusions and statements do not cover all aspects of endometriosis. They, moreover, might be biased by the personal interests of each chairperson.

Questions, conclusions and results reflect a learning process for all of us involved. Besides learning from those who have experience in web- based surveys, we suggest documenting experience- based management of endometriosis as follows. Questions should address only experience-based issues, and for mixed, experience and knowledge- based issues, the importance of experience should be decided beforehand. Questions or statements should be specific, addressing a single problem and avoiding the possibility of misinterpretation. Since questionnaires need to be limited to 20-30 min for answering to keep respondents focused, they should be limited to one or a few topics. The aim of any study should be clear by defining the type of experience of the respondents in detail, permitting them to stratify the answers by the level and type of experience. VAS scales, 0 to 10, seem appropriate, especially in web-based questionnaires. However, we fully realise that experience and knowledge- based statements are neither independent nor clearly delineated and may vary with the target population. Experience always adds knowledge, and the teacher does not only transmit knowledge but also his experience. For example, some aspects are mainly experience-based, such as suturing and knot tying, while the choice of knots is based on the knowledge of knot security. Finally, some aspects can be experience-based for surgeons and knowledge- based for non-surgeons. If the aim is to evaluate experience, it is important to stress this difference for researchers who would like to use questionnaires to document experience.

Only items that change with experience should be investigated, and questions should be precise to avoid misunderstanding.

Knowledge-based statements that do not require experience should be avoided. The same holds for opinions about the organisation of medicine and political statements. We therefore, reviewed all questions and added a third group of unclear or organisational questions.

Experience-based, evidence-based and opinion/ theory-based conclusions about endometriosis management are complementary. Clinicians usually incorporate evidence-based data in their management. This explains that none of the experience-based responses conflicted with the available evidence. Experienced-based conclusions can contribute to the difficult and unsolved problem of the extrapolation of the results of RCTs to the entire population, and they permit information for aspects that are difficult to investigate in RCTs, such as multimorbidity, rare events, absence of blinding, or multivariate clinical problems. This is important for surgery that combines multivariate decisions with relatively low numbers, making sufficiently large RCTs to permit comprehensive conclusions prohibitively difficult. As an example, for the clinician, the indication for surgery of an endometrioma is a combination of the diameter of the cyst AND pain AND radiation of pain AND age AND adhesions. During the tryout, we evaluated the feasibility of multivariate clinical questions such as pain AND diameter of the cyst. Although feasible, this was abandoned since it required too many questions. The clinician combines all these variables into a decision using knowledge and experience. Although some experience-based conclusions may differ between subspecialists, collective-experience-based conclusions for surgery are different from the grades of evidence in EBM, reflecting the judgment of a limited group of experts, some with minimal experience in surgery.

The limitations of the statements in Tables I and II should be realised. First, the analysis was limited to surgery-oriented gynaecologists or surgeons with experience in more than 50 women with endometriosis. Second, statements were considered approved if more than 75% of the experience answered the VAS scales by ≥8/10. Third, the results reflect the practice of the respondents and not necessarily the opinion of every co-author. Without discussing the individual questions of Tables I and II, most of the experience-based conclusions, indicated in red in the tables, were approved by a large majority, reflecting that their experience had been similar, thus validating the concept of collective experience. The answers to the knowledge-based statements were much more variable, reflecting the gap in knowledge between non-experts and experts, who are probably ahead in incorporating new knowledge through meetings and literature. The latter is illustrated by examples such as the lack of accurate knowledge of the efficacy of barriers in adhesion prevention and the knowledge of surgery-oriented gynaecologists about IVF and ESHRE guidelines (Becker et al., 2022). Therefore, statements with a variable answer also indicate areas where more medical education is needed. The data did not permit us to identify whether varying answers indicate differences in country, culture or specific interests such as imaging, infertility, pain management or medical therapy. Reviewing the questions, we realise that in the absence of data or experience, some answers reflect anecdotal observations such as endometriosis progressing during medical therapy or the number of severe endometriosis surgeries judged necessary for a referral surgeon. A total experience of 50 women might seem a low cut-off, but this was a compromise between reasonable and using most of the data. However, since the total individual experience does not correlate with the responses (Spearman correlation), we suggest that the opinion of younger surgeons is influenced by the experience of more experienced surgeons during congresses and live surgeries, with an update and confirmation by their own (limited) experience. Important is that experience does not mean experienced.

These experience-based results should be considered a first Bayesian prior, to being updated and refined by new investigations. Therefore, the data will be made available as SAS datasets which can be used by subsequent investigators. Many aspects remain to be explored, such as the effect of age and, gender and specific experience. However, despite all limitations, many conclusions are the best evidence we have today. This is especially important for endometriosis since the evidence is scarce in the absence of a non-invasive diagnosis, the lack of blinding of many hormonal medical therapies and the complexity of surgery.

The conclusions compare well with the recent ESHRE guidelines (Becker et al., 2022). Although a complete discussion is beyond this manuscript’s scope, we are unaware of a single contradiction. This is not surprising since solid evidence of EBM was probably already incorporated into experience- based medicine. Experience-based conclusions will complement guidelines when limited to recommendations based on indirect evidence and common sense.

## Appendices Questionnaires 1-2: scan QR

https://qrco.de/beMd5f

**Figure:**
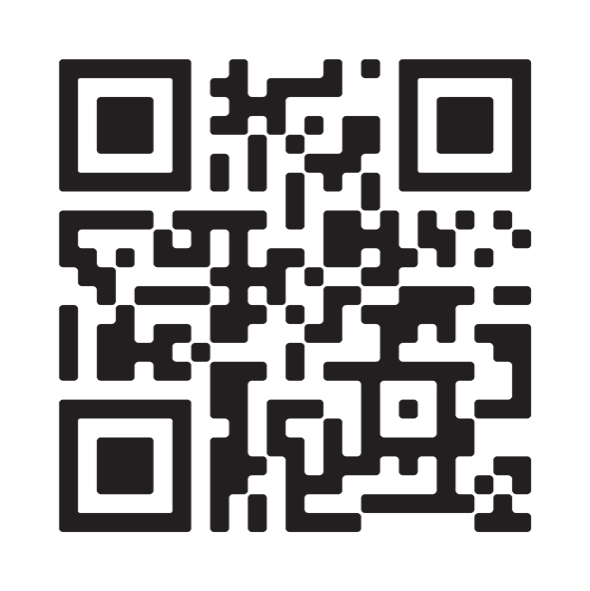


https://qrco.de/beMdBR

**Figure:**